# Spinal cord haemangiosarcoma in one dog – Case report

**DOI:** 10.17221/60/2023-VETMED

**Published:** 2023-10-30

**Authors:** Maria Kuricova, Jakub Fuchs, Tomas Liptak, Filip Korim, Natalia Surin Hudakova, Bisal Bhattarai, Zoltan Kerekes, Viera Revajova

**Affiliations:** ^1^Small Animal Clinic, University of Veterinary Medicine and Pharmacy in Kosice, Kosice, Slovak Republic; ^2^Department of Morphological Disciplines, University of Veterinary Medicine and Pharmacy in Kosice, Kosice, Slovak Republic; ^3^Department of Microbiology and Immunology, University of Veterinary Medicine and Pharmacy in Kosice, Kosice, Slovak Republic; ^4^Moscow State Academy of Veterinary Medicine and Biotechnology MVA named after K. I. Skryabin, Ulitsa Akademika Skryabina, Moscow, Russia; ^5^Zoltan Kerekes, VetScan, Veterinary Imaging Center, Budapest, Hungary

**Keywords:** canine, intramedullary, paralysis, tumour

## Abstract

A 5-year-old intact female Shih Tzu was presented with acute onset of hind leg paralysis. The neurologic examination revealed severe T3-L3 myelopathy. The differential diagnoses included degenerative, anomalous, traumatic, inflammatory, vascular, metabolic, and neoplastic changes. The results of the paraclinical examinations and diagnostic imaging narrowed the list of differential diagnoses and, along with the patient’s deteriorating condition, led to the owner’s decision to euthanise the dog. The histologic findings of the spinal cord specimens indicated a tumour of the blood vessels formed by the proliferation of endothelial cells, which may present as either capillary or cavernous structures. In this case, the tumour was a capillary-type haemangiosarcoma. The primary site of proliferation could not be determined in this case because no mass formation was noted while performing the necropsy.

Spinal cord tumours in dogs are classified as primary and secondary ones, depending on their location in the spinal cord, as well as extradural (most common – 50%), intradural-extramedullary (30%), and intramedullary (rare – 15%). Primary spinal tumours are relatively rare in most domestic animal species with the exception of dogs and, to a lesser extent, cats. In dogs, the most common spinal cord tumours are meningiomas that mainly affect the cervical segments. Intramedullary spinal tumours in dogs are also rare, and these are mainly glial cell tumours, astrocytomas, oligodendrogliomas, undifferentiated sarcomas, ependymomas, and choroid plexus papillomas. Primary intradural extramedullary tumours are usually diagnosed in young dogs (6 months to 3 years). They are classified as neuroepithelioma, ependymoma, medulloepithelioma, nephroblastoma, and spinal blastoma. The most common secondary tumour affecting the spinal cord of dogs is a haemangiosarcoma. Haemangiosarcomas and lymphomas tend to invade the spinal cord intramedullary, and secondary metastatic disease has also been reported in the form of mammary adenocarcinoma and malignant melanoma (Drost et al. 1996; Le Couter and Withrow 2007; Bagley 2010; Brewer et al. 2011; Pancotto et al. 2013; Higgins et al. 2017).

## Incidence of spinal tumours in the dog

The most commonly diagnosed extradural tumours in dogs are primary malignant bone tumours (osteosarcoma, chondrosarcoma, fibrosarcoma, haemangiosarcoma, haemangioendothelioma, and myeloma) and tumours that metastasise to bone and soft tissue. Secondary tumours affecting canine vertebrae include numerous primary tumour types. Metastatic extradural neoplasms are uncommon in dogs (Lewis et al. 1991; Le Couter and Withrow 2007; Morgan et al. 2007; Bagley 2010).

Meningiomas and peripheral nerve sheath tumours are the most common intradural-extramedullary neoplasms in dogs. These tumours occur most commonly in older dogs of both sexes. Meningiomas are considered the most common type of tumour affecting the spinal cord (Le Couter and Withrow 2007; Petersen et al. 2008; Besalti et al. 2016). Less common spinal cord tumours are intradural-extramedullary tumours of the spinal cord affecting young dogs (Ribas 1990; Luttgen 1992; Le Couter and Withrow 2007; Brewer et al. 2011).

Intramedullary spinal tumours in dogs are rare. Granulomatous meningoencephalomyelitis can sometimes occur as a primary intramedullary spinal cord lesion. We can also encounter intramedullary metastases in the spinal cord of dogs with systemic malignancy (Waters and Hayden 1990; Le Couter and Withrow 2007; Lipitz et al. 2011; Sloma et al. 2015; Saitoh et al. 2021).

## Clinical signs of spinal tumours in dogs

Extramedullary neoplasms of the spinal cord usually grow slowly and gradually compress the spinal cord. Symptoms of spinal cord dysfunction worsen over weeks or months. Occasionally, the acute onset of clinical signs may be associated with haemorrhage or ischemia related to the neoplasia. Intramedullary tumours, which can grow faster, are characterised by a higher incidence of ischemia, necrosis, and haemorrhage. Clinical signs associated with spinal cord tumours usually reflect the location of the neoplasia and are often indistinguishable from symptoms caused by other transverse myelopathies at the same site. Extreme spinal hyperesthesia is one of the clinical manifestations of meningeal tumours and spinal nerve roots or nerve sheets tumours, resulting in various degrees of discomfort.

Neurological deficits (e.g., paresis) may not be apparent at first and may be intermittent (i.e., exacerbated by exercise). Neurological function deteriorates caudally from the lesion over time. Intradural-extramedullary tumours can result in chronic, intermittent clinical signs, and spinal hyperesthesia.

Damage to the brachial or lumbar intumescence is usually accompanied by lameness, a non-weight bearing gait, neurogenic muscle atrophy, and weakening of the spinal reflexes. Rarely, unilateral spinal cord compression can cause neurologic deficits in the contralateral limb.

In contrast, intramedullary spinal cord tumours usually quickly exacerbate the neurologic dysfunction, with hyperesthesia being rarely associated with these types of tumours (Waters and Hayden 1990; Braund 2003; Le Couter and Withrow 2007; Petersen et al. 2008; North and Banks 2009; Valentim et al. 2016).

## Diagnosis of spinal tumours in dogs

The diagnosis of neoplasia affecting the spinal cord requires a systematic approach. The diagnosis is based on the collection and evaluation of a minimal database that includes appropriate blood tests (haemogram, biochemical profile) and chest radiographs to visualise the primary or metastatic neoplasia.

The next step is to perform radiographs of the spine. The collection and analysis of the cerebrospinal fluid and myelography or advanced imaging are other necessary steps for diagnosis (Drost et al. 1996; Le Couter and Withrow 2007; Petersen et al. 2008; Auger et al. 2021).

## Therapy of spinal tumours in dogs

For dogs, there are only a limited number of therapeutic options for spinal cord tumours today. The appropriate therapy depends on the location, extent, and histologic type of the tumour. The immediate goal of therapy is to alleviate the detrimental effects of the existing spinal cord compression. This can be achieved with medication (e.g., glucocorticoids) or surgery. Surgical therapy may allow the complete removal or cytoreduction and biopsy of the neoplasia. If the complete removal of the tumour is not possible, recurrence and adjunctive therapy should be considered. The development of advanced neurosurgical techniques and the introduction of new biopsy techniques have improved the therapeutic outcome in many cases. An accurate biopsy diagnosis is especially crucial in lymphoma, as lymphomas of the spinal cord can be successfully treated with chemotherapy or radiotherapy alone or in combination (Le Couter and Withrow 2007; Bagley 2010; Paek et al. 2015; La Rue et al. 2018).

## Prognosis of spinal tumours in dogs

Some of the data concerning the long-term follow-up and outcomes in dogs and cats with spinal neoplasms are limited. The prognosis depends on the surgical resectability, histologic type, location, and severity of clinical signs. Extradural metastatic tumours usually have a poor prognosis, and only palliative treatment us usually attempted. The removal of the affected vertebra (spondylectomy) has been described in many studies, and it may be attempted in the lumbar region. Intradural extramedullary tumours can occasionally be completely resected, in which case the prognosis is considered good. In one study, five of the nine dogs studied survived more than 6 months after surgical resection of the meningioma. Of these five dogs, one was still alive 3 years after surgery. Few data are available on undergoing radiation or chemotherapy of spinal cord tumours in animals. Few studies describe the treatment of intramedullary tumours in dogs with radiation therapy. The results in these cases suggest that canine spinal cords tolerate this type of therapy well and that the clinical signs can be alleviated for more than one year (Siegel et al. 1996; Levy et al. 1997; Ueno et al. 2006; Bagley 2010).

## Case description

**Figure 1:**
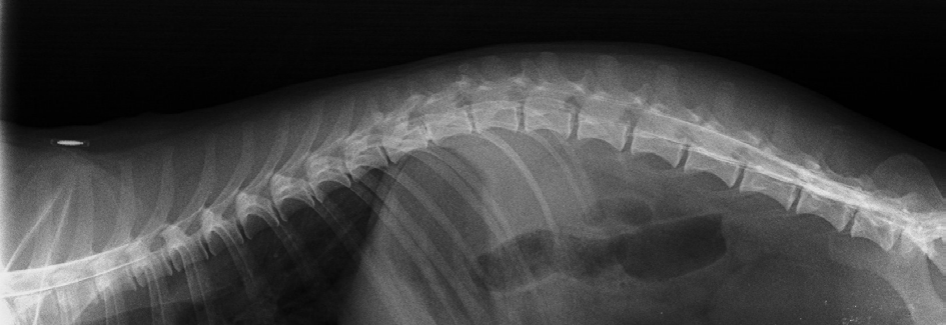
Anonymised myelogram of the investigated dog Impaired filling of the contrast agent in the vertebral canal indicates the marked enlargement of the spinal cord in the thoracic and lumbar segments

A 5-year-old female Shih Tzu weighing 3.7 kg was presented with paralysis of the hind legs, urination and defecation on a mat, and consistent water and food intake for three days. The physical examination revealed a good general condition, the dog was paraplegic, the spinal reflexes were normal on the front legs, increased on both pelvic legs except for the flexor reflex, superficial sensation on the pelvic legs was absent bilaterally, deep pain perception was absent, musculocutaneous reflex caudal of T2 was absent, and the spine was painless on palpation. The diagnosis was T3-L3 myelopathy. The haematological examination revealed only mild anaemia (erythrocytes 4.6 × 10^12^/l; reference range 4.95–7.87 × 10^12^/l); the biochemical examination revealed a slightly increased alkaline phosphatase activity (ALP 2.0 μkat/l, reference range 0.02–1.90 μkat/l). The radiographic examination showed a markedly filled urinary bladder, gas in the stomach and colon, rounded edges of the spleen, calcification of the intervertebral disc between T11-T12, and a slight narrowing of the disc space between T12-T13. The ultrasonography of the abdominal cavity revealed no pathologic changes in addition to those noted on radiography (splenomegaly, ileus). The myelography showed a filling defect within the spinal canal from T5 to L3 (Figure 1).

**Figure 2:**
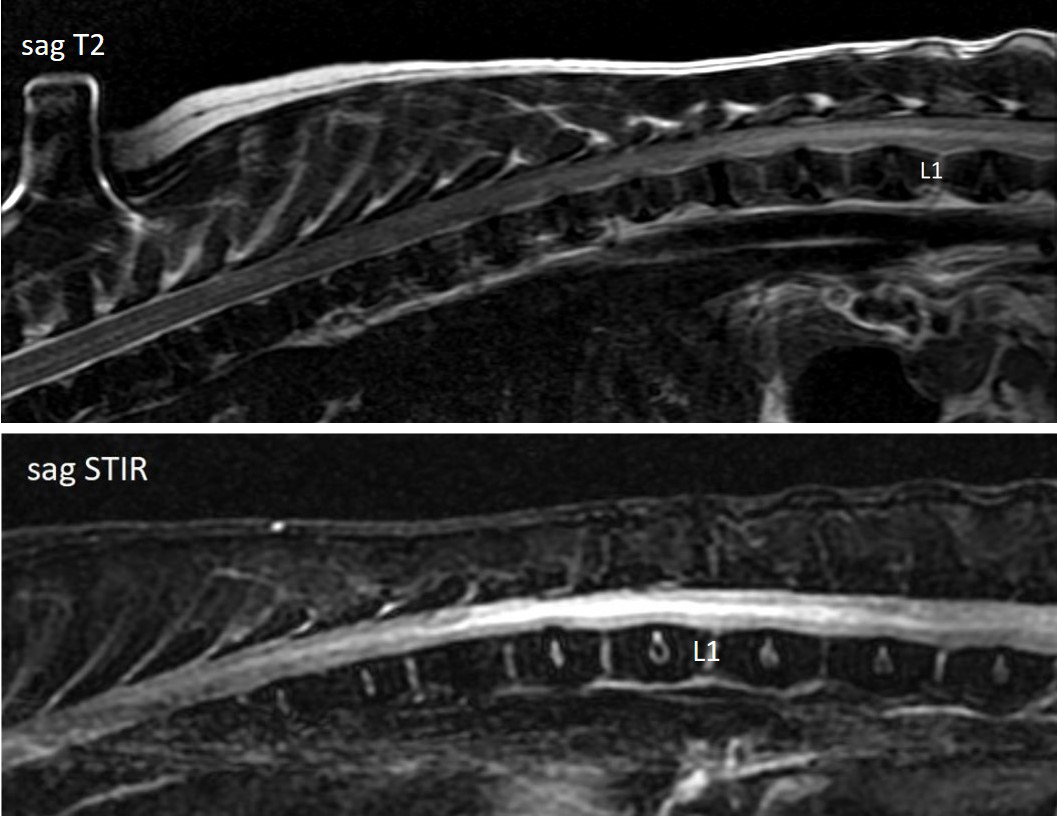
Magnetic resonance STIR and T2w images of the investigated dog The MRI revealed a heterogeneous spinal cord at the T5-T6 level with a T2 hypointense mass (epidural and subdural), homogeneous areas with high T2 signal intensity in the spinal cord parenchyma at the T11 to L3 level. On the T2*GRE imaging, these structures were significantly hypointense, and hyperintense on the STIR. The final evaluation revealed severe intramedullary and perispinal lesions with intramedullary, subdural, and epidural haemorrhage and presumed myelomalacia

For the magnetic resonance imaging (MRI), the sequences Sag T2 FSE, Sag STIR, Sag T1 FSE, dorsal T2 FSE, transverse T2 FSE, and transverse T2*GRE were used. The MRI showed a rather heterogeneous spinal cord parenchyma between vertebrae T5 and L6, and the dorsal and ventral cerebrospinal fluid (CSF) spaces were not visible at this location. On the T2-w sequences, a mass was found in the right dorsolateral, right lateral, right ventrolateral epidural, and subdural spaces with very low signal intensity; this material was homogeneous. In the spinal cord, a sharply defined area of high signal intensity was found in the middle of the spinal cord in the T11-L3 region in the T2w sequences. In the T2*GRE sequence, sharply demarcated, homogeneous areas of very low signal intensity were found in the spinal cord at the previously mentioned locations. The previously mentioned epidural subdural mass also had very low signal intensity in the T2*GRE sequence. Minimal to mild spinal cord compression was present due to disc bulging (Figure 2).

The images were obtained using a GE Signa Explorer 1.5 T with a slice thickness of 3 mm, a gap of 0.3 mm, a USCT234 coil, and a posterior array. The CSF examination revealed a slightly yellowish appearance macroscopically; the laboratory noted a greater number of erythrocytes and increased protein content. The cytologic examination with a cytocentrifuge (cytospin) revealed no evidence of tumour cells.

**Figure 3:**
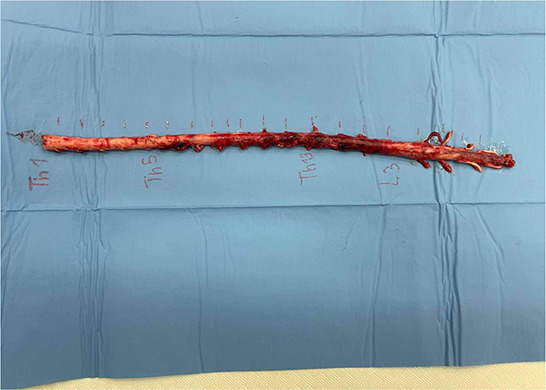
Macroscopic image of the affected spinal cord, which is markedly haemorrhagic, oedematous and myelomalacic

At the owner’s request for euthanasia and the histologic examination of the spinal cord specimens, a dorsal laminectomy was performed through all the segments of the thoracic and lumbar spine after humane euthanasia. The spinal cord was markedly malacic with epidural, subdural, and intramedullary haemorrhages that correlated with the MRI findings (Figure 3).

**Figure 4:**
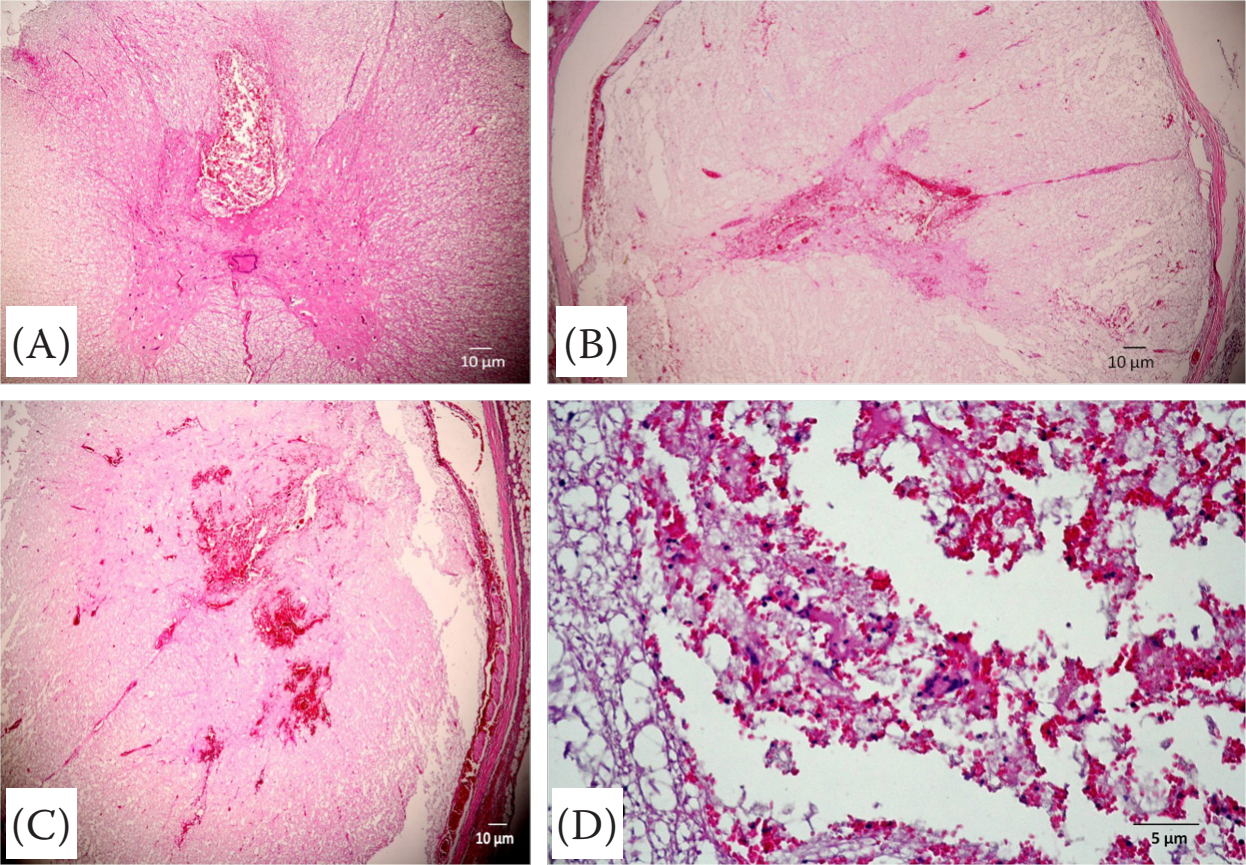
Histological findings of the affected spinal cord (A) An oval-shaped lesion in the parenchyma of the spinal cord at the level of the T5 vertebra (H&E staining, bar** =** 10 μm). (B) Oedema, extravasations and multiplicity of capillaries in the spinal cord region at the level of the T10 vertebra (H&E staining; bar** =** 10 μm). (C) Multifocal extensive to coalescing foci of bleeding and the presence of newly formed vessels in the parenchyma and around the spinal cord at the level of L1 vertebra (H&E staining; bar** =** 10 μm). (D) Endothelial proliferation with capillary formation in the devastated parenchyma of the spinal cord (H&E staining; bar** =** 5 μm)

**Figure 5:**
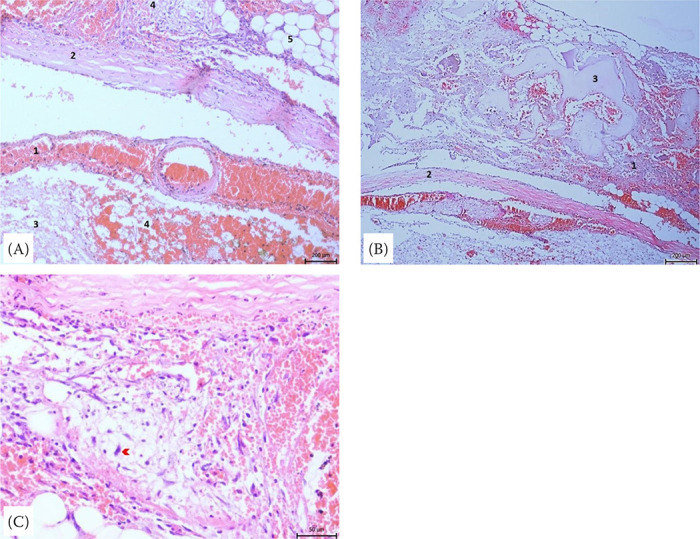
Histological finding of the diagnosed haemangiosarcoma (A) Proliferation of endothelial cells (4) in the white matter of the spinal cord (3) under haemorrhagic leptomeninx (1), above the dura mater (5), and interstitially in adipose tissue at the level of L1 vertebra. (B) Spreading of the tumour (1) in the perispinal area supradurally (2) with the destruction of the chondroid tissue (3) at the level of L1 vertebra. (C) Anisocytosis, anisokaryosis, and atypical mitosis (red arrow) of endothelial cells forming irregular walls of blood vessels and cavernae in haemangiosarcoma

**Figure 6:**
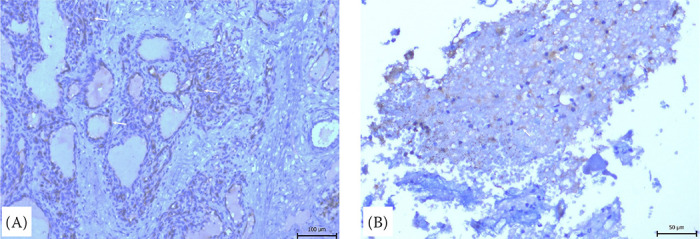
Immunohistochemistry used mouse-antihuman CD 31 antibody staining on the endothelial cells in the subcutaneous capillary haemangioma as a positive control CD 31 antibody staining on the endothelial cells (A) in the subcutaneous capillary haemangioma as a positive control; Bar = 100 μm, and (B) on the canine haemangiosarcoma at the level of the L1 vertebra; Bar = 50 μm

The spinal cord specimens from segments T5-T6, T10-T11, and L1-L2 were sent for a histopathological examination. They were processed by routine histological procedure (haematoxylin-eosin – H&E) (Figure 4A–D, Figure 5A–C) and by immunohistochemistry (specimen from L1 level, CD 31) (Figure 6).

In the cranially located specimen (T5-T6), the central canal was filled with a pink homogeneous fluid, between the dorsal column of the spinal cord in the grey and white matter, extensive ovoid haemorrhages were visible, relatively limited, without inflammatory infiltration, violating the integrity of the nervous tissue of the spinal cord. There were small capillaries with blood stasis in the surrounding tissue of the spinal cord. In the section from T10-T11, the pre-existing disruption of the spinal cord parenchyma at the site of haemorrhage was less pronounced than in the specimen from the level of T5. A greater number of capillaries, extravasations, and oedema of the spinal cord parenchyma was observed.

These changes were found not only in the white and grey matter, but also in the spinal cord meninges. The same diffuse haemorrhage was also present in the spinal cord section at the level of L1-L2, but vessels were also detected above the dura mater.

The endothelium was spindle-shaped, and pathologic mitoses were rarely present in the nuclei. There was a focal neutrophilic infiltrate, neurons with hemosiderin accumulation, which was present free as well as in the siderophages in the areas of extravasations. The diagnosis was hemangiosarcoma.

## DISCUSSION AND CONCLUSION

Haemangiosarcomas are neoplasms of vascular endothelial origin commonly diagnosed in dogs, with the skin being a strong predilection site in addition to a very common visceral form. Visceral haemangiosarcomas have no predilection site and usually involve the spleen, right atrium, and liver. They are considered highly metastatic tumours, with a disseminated neoplasia found in more than 80% of cases (Hargis et al. 1992; Smith 2003). In our case, we found no evidence of a primary tumour in the other organs. Aschenbroich et al. (2014) described a visceral and disseminated haemangiosarcoma in a paraparetic golden retriever with involvement of the spinal cord, heart, and lungs (Aschenbroich et al. 2014). In most cases, the coexistence of neoplasms in multiple organs makes it difficult to distinguish between a multicentric or metastatic process. In a multicentre, a retrospective case series study by Mallol et al. (2022), vertebral haemangiosarcomas were segmental, poorly emarginated, polyostotic, and very aggressive lesions that invaded the thoracic spinal canal and paraspinal tissue. Epidural haemangiosarcomas were single, well-defined lesions in the thoracolumbar and/or lumbar regions. Intramedullary haemangiosarcomas were cervical, had a metastatic origin, and were frequently (3/4) accompanied by intracranial lesions (Mallol et al. 2022). We consider that the haemangiosarcoma in the spinal cord in our case was a primary and not metastatic type, although a metastatic origin cannot be completely excluded.
